# Supplementary material for: An offline-first electronic health record for vulnerable populations: A mixed-methods feasibility study
Source: PLOS Digit Health. 2026 Feb 13;5(2):e0001204. doi: 10.1371/journal.pdig.0001204 (PMC12904448; doi:10.1371/journal.pdig.0001204)
Supplement: S1 Appendix — This appendix contains all of the definitions used in our feasibility study. These definitions were built off standard definitions in the literature. (DOCX) [file pdig.0001204.s001.docx]

**S1 Appendix**: Standard Feasibility Definitions

**Hikma Health EHR System:** This refers solely to the EHR itself. References to its usability, workflows, or software malfunctions.

**Implementation Package:** This refers to the training, guidance, and technical support used to integrate the EHR into the care provided at the clinic.

**Acceptability:** The extent to which the Hikma Health EHR is judged as suitable, satisfying, or attractive to participants.

- Will be assessed by satisfaction, perceived appropriateness, including ease of use of the EHR innovation.

**Practicality:** The extent to which the Hikma Health EHR is implemented with intended participants using existing means and resources.

- Will be assessed through hardware availability, electricity and internet demands, and capacity for equipment maintenance and replacement.

**Integration:** The extent to which the Hikma Health EHR is integrated within the organization's existing system.

- Will be assessed by evaluating how the Hikma Health EHR system is incorporated into daily practice, perceived sustainability, gauging perceptions of availability of a supportive working environment, and assessing integration with the existing procurement, maintenance structures and processes (including engineering capacity)

**Limited Effectiveness:** Does the Hikma Health EHR show potential for improving operational metrics or patient outcomes.

- Will be assessed by participant perspectives on how the Hikma Health EHR affects overall clinical or patient outcomes such as increased clinical efficiency, reduced clerical time, reduced medical errors, and overall patient outcomes. This will be assessed by directly asking participants to estimate metrics such as number of patients seen before and after EHR implementation
