# Supplementary material for: An offline-first electronic health record for vulnerable populations: A mixed-methods feasibility study
Source: PLOS Digit Health. 2026 Feb 13;5(2):e0001204. doi: 10.1371/journal.pdig.0001204 (PMC12904448; doi:10.1371/journal.pdig.0001204)
Supplement: S2 Appendix — This appendix has the interview guide that was used to conduct the in-depth interviews with healthcare workers and administrators. (DOCX) [file pdig.0001204.s002.docx]

**S2 Appendix:** Interview Guide for Hikma Health's Electronic Health Record (EHR) Project

Experience: Questions to understand the participants' level of experience and training with medical records and Hikma Health's EHR.

1. How have you stored and recorded patient data before the HH EHR?
2. What did you like about that system?
3. What did you dislike about that system?
4. What system are you currently using? Do you use different systems for different patients or circumstances?
5. Did you receive training on how to use Hikma Health’s EHR? What was the training like and who conducted it? (duration and timing) Was it adequate? How long was this training?
6. When you were learning to use Hikma Health’s EHR, what aspects were difficult? What was easy? (Interviewer notes: Consider terminology, workflow, data access, data entry, and patient search).
7. How long did it take for you to feel comfortable using Hikma Health’s EHR? Why?

Acceptability: The extent to which Hikma Health’s EHR is considered adequate, satisfactory, or appealing to participants. It will be evaluated through perceived satisfaction and convenience, including the ease of use of the EHR innovation.

1. What has been your experience using Hikma Health’s EHR?
2. Have you encountered any difficulties using Hikma Health’s EHR?
3. What are the limitations of Hikma Health’s EHR?
4. What benefits have you found using Hikma Health’s EHR?

Practicality: The extent to which Hikma Health’s EHR is implemented with participants using existing means and resources. It will be evaluated through hardware availability, electricity and internet demands, and the maintenance and replacement capacity of equipment.

1. Have you had any technical difficulties using Hikma Health’s EHR?
2. Do you use a tablet, a mobile phone, or both when using Hikma Health’s EHR?
3. For any device: What has been your experience using this device for the EHR? What successes or challenges did you face? Were you able to resolve these problems?
4. What has been your experience syncing Hikma Health’s EHR?

Integration: The extent to which Hikma Health’s EHR is integrated into the organization’s existing system. It will be evaluated considering how the EHR is incorporated into daily practices, perceived sustainability, measuring perceptions of a favorable work environment, and assessing integration with existing acquisition and maintenance structures (including engineering capacity).

1. How has Hikma Health’s EHR integrated into your work environment?
2. Could you walk me through your workday and how you integrate and use Hikma Health’s EHR?
3. In what other areas of your daily work could Hikma Health’s EHR help even more?
4. Do you believe you have the necessary support to effectively use Hikma Health’s EHR? If so, describe it. If not, what would you like?
5. Do you think you have the necessary IT support to use Hikma Health’s EHR? Do you think you will need more IT support in the future?
6. Do you think the use of Hikma Health’s EHR will be sustainable over the years in this clinic? Why or why not?
7. Do you have any ethical concerns with using Hikma Health’s EHR (prompt: privacy, bias)?

Limited Effectiveness: Measured by how Hikma Health’s EHR shows potential to improve operational metrics or patient outcomes. It will be evaluated based on participants’ perspectives on how Hikma Health’s EHR affects clinical or patient outcomes, such as increased clinical efficiency, reduced administrative time, reduced medical errors, and overall patient outcomes.

1. Overall, how do you think Hikma Health’s EHR has affected your ability to provide effective medical care?
2. Has using Hikma Health’s EHR changed your typical interaction with patients?
3. Before using Hikma Health’s EHR, on average, how long did it take to complete a patient encounter? How long does it take now?
4. How do these results compare to other medical record systems you have used?
5. Do you think Hikma Health’s EHR has had an effect on patient outcomes? Which outcomes?
6. Do you think there were better or worse results for certain patients or patients with specific illnesses?
7. How do you think using Hikma Health’s EHR has affected medical errors?
8. Do you think it is better or worse than the paper system?
9. Is there anything else you would like to share about your experience with Hikma Health’s HER
