# Supplementary material for: An offline-first electronic health record for vulnerable populations: A mixed-methods feasibility study
Source: PLOS Digit Health. 2026 Feb 13;5(2):e0001204. doi: 10.1371/journal.pdig.0001204 (PMC12904448; doi:10.1371/journal.pdig.0001204)
Supplement: S3 Appendix — This questionnaire was given to every in-depth interview participant to understand their experience that could have informed their interview. (DOCX) [file pdig.0001204.s003.docx]

**S3 Appendix:** Hikma Health EHR Background Questionnaire

1. Gender:
   1. Man
   2. Woman
   3. Transgender
   4. Non-binary/non-conforming
   5. Prefer not to respond
2. Age in years
3. What is your primary role?
   1. Physician
   2. Nurse
   3. Healthcare assistant or tech
   4. Administrator
   5. In-take Staff
   6. Pharmacist
   7. Engineer
   8. Other:
4. Number of years in profession:
5. Number of years working at current clinic:
6. Number of months using Hikma Health EHR:
