# Supplementary material for: An offline-first electronic health record for vulnerable populations: A mixed-methods feasibility study
Source: PLOS Digit Health. 2026 Feb 13;5(2):e0001204. doi: 10.1371/journal.pdig.0001204 (PMC12904448; doi:10.1371/journal.pdig.0001204)
Supplement: S4 Appendix — This is the Standard for Quality Improvement Reporting Excellence (SQUIRE 2.0) checklist, used to ensure that this study adhered to the standards of mixed-methods studies. (DOCX) [file pdig.0001204.s004.docx]

S4 Appendix: SQUIRE checklist

**Revised** **Standards for Quality Improvement Reporting Excellence (SQUIRE 2.0) September 15, 2015**

| **Text Section and Item**  **Name** | **Section or Item Description** |
| --- | --- |
| **Notes to authors** | - The SQUIRE guidelines provide a framework for reporting new   knowledge about how to improve healthcare   - The SQUIRE guidelines are intended for reports that describe [system](#_bookmark13) level work to improve the quality, safety, and value of healthcare, and used methods to establish that observed outcomes were due to the [intervention(s).](#_bookmark8) - A range of approaches exists for improving healthcare. SQUIRE may be adapted for reporting any of these. - Authors should consider every SQUIRE item, but it may be inappropriate or unnecessary to include every SQUIRE element in a particular manuscript. - The SQUIRE Glossary contains definitions of many of the key words in SQUIRE. - The Explanation and Elaboration document provides specific examples of well-written SQUIRE items, and an in-depth explanation of each item. - Please cite SQUIRE when it is used to write a manuscript. |
| **Title and Abstract** |  |
| **1. Title** | Indicate that the manuscript concerns an [initiative](#_bookmark6) to improve healthcare (broadly defined to include the quality, safety, effectiveness, patient- centeredness, timeliness, cost, efficiency, and equity of healthcare)  **The title specifies an initiative (an offline first electronic health record), the overall assessment (feasibility study) and specific patient populations** |
| **2. Abstract** | 1. Provide adequate information to aid in searching and indexing 2. Summarize all key information from various sections of the text using the abstract format of the intended publication or a structured summary such as: background, local [problem,](#_bookmark10) methods, interventions,   results, conclusions  **The abstract contains information on the background, context, methods, interventions, results and conclusions about the study in an unstructured format.** |
| **Introduction** | *Why did you start?* |
| [**3. Problem**](#_bookmark10) [**Description**](#_bookmark10) | Nature and significance of the local [problem](#_bookmark10)  **The problem and its contributing factors are both defined and quantitatively assessed by previous research.** |
| **4. Available knowledge** | Summary of what is currently known about the [problem,](#_bookmark10) including relevant previous studies.  **A summary of previous work on electronic health records for limited resources settings is discussed including the findings of a systematic review as well as references to specific landmark articles.**  **A summary of other previous EHRs designed for low resource settings is also discussed and previous literature.** |

| **5.** [**Rationale**](#_bookmark12) | Informal or formal frameworks, models, concepts, and/or [theories](#_bookmark14) used to explain the [problem,](#_bookmark10) any reasons or [assumptions](#_bookmark0) that were used to develop the [intervention(s),](#_bookmark8) and reasons why the [intervention(s)](#_bookmark8) was  expected to work  **The rationale is discussed including the lack of offline first electronic health records and the needed to better understand the feasibility of a solution that has already been implemented.** |
| --- | --- |
| **6. Specific aims** | Purpose of the project and of this report  **The purpose of the project is clearly stated in the final paragraph of the introduction.** |
| **Methods** | *What did you do?* |
| **7.** [**Context**](#_bookmark1) | Contextual elements considered important at the outset of introducing the [intervention(s)](#_bookmark8)  **Specific context is given about the HH EHR as well as context on each clinic that the EHR was implemented in.** |
| **8.** [**Intervention(s)**](#_bookmark8) | 1. Description of the [intervention(s)](#_bookmark8) in sufficient detail that others could reproduce it 2. Specifics of the team involved in the work   **The intervention is well described as well as each authors role in the research through the explicit naming of initials** |
| **9. Study of the Intervention(s)** | 1. Approach chosen for assessing the impact of the [intervention(s)](#_bookmark8) 2. Approach used to establish whether the observed outcomes were due to the [intervention(s)](#_bookmark8)   **We discuss the overall approach through a feasibility study and define each measure of feasibility based on prior research in implementation science. We also discuss the specific qualitative framework we used to conduct this research.** |
| **10. Measures** | 1. Measures chosen for studying [processes](#_bookmark11) and outcomes of the [intervention(s),](#_bookmark8) including rationale for choosing them, their operational definitions, and their validity and reliability 2. Description of the approach to the ongoing assessment of contextual elements that contributed to the success, failure, efficiency, and cost 3. Methods employed for assessing completeness and accuracy of data   **We discuss the overall mixed methods approach using quantitative and qualitative data. While the qualitative data was much stronger than the quantitative, we discuss both methods and the instruments used to collect all data. We discuss how all measures are based on the feasibility framework that was initiated and based on the definitions of feasibility.** |
| **11. Analysis** | 1. Qualitative and quantitative methods used to draw [inferences](#_bookmark5) from the data 2. Methods for understanding variation within the data, including the   effects of time as a variable  **We discuss both the qualitative and quantitative instruments used. We collected all the data at once and did not discuss the effect of time as a variable.** |
| **12. Ethical**  **Considerations** | [Ethical aspects](#_bookmark2) of implementing and studying the [intervention(s)](#_bookmark8) and how they were addressed, including, but not limited to, formal ethics review and potential conflict(s) of interest  **We report both IRBs that the study was submitted to and approved by, we also discuss conflicts of interest in the author statement at the end.** |
| **Results** | *What did you find?* |
| **13. Results** | 1. Initial steps of the [intervention(s)](#_bookmark8) and their evolution over time (*e.g.*, time-line diagram, flow chart, or table), including modifications made to the intervention during the project 2. Details of the [process](#_bookmark11) measures and outcome 3. Contextual elements that interacted with the [intervention(s)](#_bookmark8) 4. Observed associations between outcomes, interventions, and relevant contextual elements 5. Unintended consequences such as unexpected benefits, problems, failures, or costs associated with the [intervention(s).](#_bookmark8) 6. Details about missing data   **We discuss the main modification made to the HH EHR at the Nueva Vida Clinic Site and the impacts the issue had with synching made on clinic care before the changes were made. We provide both quantitative and qualitative data showing the effect of the HH EHR on each category of feasibility as it was previously defined. These are also put in context of the two different clinics. We did not have any missing data to report.** |
| **Discussion** | *What does it mean?* |
| **14. Summary** | 1. Key findings, including relevance to the [rationale](#_bookmark12) and specific aims 2. Particular strengths of the project   **We discuss the overall findings of this project and how it met the specific aims of feasibility**. |

| **15. Interpretation** | 1. Nature of the association between the [intervention(s)](#_bookmark8) and the outcomes 2. Comparison of results with findings from other publications 3. Impact of the project on people and [systems](#_bookmark13) 4. Reasons for any differences between observed and anticipated outcomes, including the influence of [context](#_bookmark1) 5. Costs and strategic trade-offs, including [opportunity costs](#_bookmark9)   **We comment on the results and how they differ between intervention sites and how are findings relate to previous work in this field. We were not explicitly able to discuss the opportunity costs of this work, but do discuss a comparison to paper charting and its impact on medical errors.** |
| --- | --- |
| **16. Limitations** | 1. Limits to the [generalizability](#_bookmark3) of the work 2. Factors that might have limited [internal validity](#_bookmark7) such as confounding, bias, or imprecision in the design, methods, measurement, or analysis 3. Efforts made to minimize and adjust for limitations   **Limitations and biased are discussed in their own section of the discussion including issues with generalizability (feasibility not effectiveness study) as well as concerns for bias based on the influx of resources from this study.** |
| **17. Conclusions** | 1. Usefulness of the work 2. Sustainability 3. Potential for spread to other [contexts](#_bookmark1) 4. Implications for practice and for further study in the field 5. Suggested next steps   **The conclusion covers the implications as well as the successes and drawbacks of this intervention. This includes sustainability and implications for further practice. It also discusses limitations and biases in this work as well as suggesting next steps for further improvement.** |
| **Other information** |  |
| **18. Funding** | Sources of funding that supported this work. Role, if any, of the funding organization in the design, implementation, interpretation, and reporting  **Funding is discussed at the end in the Competing Interest Statement, including that Hikma Health funded this research, but authors are volunteers and not paid or compensated in anyway for their work.** |
