# Supplementary material for: An offline-first electronic health record for vulnerable populations: A mixed-methods feasibility study
Source: PLOS Digit Health. 2026 Feb 13;5(2):e0001204. doi: 10.1371/journal.pdig.0001204 (PMC12904448; doi:10.1371/journal.pdig.0001204)
Supplement: Attachment — Submitted filename: REVIEW PLOS 18 April 2025 Criteria for publication.docx [file pdig.0001204.s005.docx]

**Criteria for publication**

In addition to being ethically conducted and scientifically valid, research published in *PLOS Digital Health* should fulfill each of the following criteria:

1. Summary of the research

In your own words, summarize the main research question, claims, and conclusions of the study. Provide context for how this research fits within the existing literature. Discuss the manuscript’s strengths and weaknesses and your overall recommendation.

The implementation of the offline electronic health record system particularly in resource poor environments, displaced or migratory patients’ and those that may be in rural and remote contexts as a critical issue facing developed and developing nations globally.

Research regarding EHR challenges and strengths particularly with offline ecosystems is an innovative strategy that could enhance the capacity of communities, clinicians and patients alike with appropriate governance.

This study resulted in more efficient management of patient records, reduced paperwork, and improved communication among healthcare providers. Clinicians reported feeling more confident in their ability to provide quality care to their patients, as they had easy access to patient information and medical history at their fingertips. Additionally, the offline system allowed for continuous access to patient data even in areas with limited internet connectivity, ensuring continuity of care.

Overall, the implementation of the offline electronic health record system was successful in improving clinic operations and clinicians' ability to deliver high-quality healthcare services to patients in resource-limited settings and offline options for locations that have challenges with technology and cyberinfrastructure capacity issues.

***2. Examples and evidence Major issues***

**Major issues** must be addressed in order for the manuscript to proceed. Focus on what is essential for the current study, not the next step in the research. Put these items in a list and be as specific as possible.

Nil major issues.

**Minor issues**

Mention additional things the authors should do to improve the manuscript. Typically these will be changes that would not affect the overall conclusions.

**Utility for the broader community or for non-clinical patient /consumer groups regarding how such an EHR may be relevant to displaced communities:**

#Research has primarily focused on barriers at the individual level, and considering if examining the broader organizational/systemic factors and responsibilities for example of the government or how the current health system where the study was conducted uses EHR (or perhaps lack of the EHR) that contribute to the challenges in the health information system identified.

There may have been a lack of infrastructure regarding organizational/systemic barriers to allow for prior successful implementation of healthcare technologies in these locations but creating a picture of prior systems or challenges historically may give readers who have not worked in these settings a better understanding.

Consider expanding perhaps systemic issues from other nations (deidentifying countries and systemic global commentary) could be helpful in the background section. This may create an opportunity for further local and system health care work/projects to support the patient’s and clinicians and have the study be a vehicle for highlighting any system issues specific to the study location.

For example, regarding biases considering the investment capability in both individual and organizational interventions, may be helpful to communicate the usefulness of the EHR in terms of benefits re: successful implementation of healthcare technologies to highlight global health access issues.

#One key advantage of EHRs in non-hospital settings is the ability to centralize patient information, making it easier for healthcare providers to access and share data. This can lead to better coordination of care, reduced medical errors, and improved patient outcomes. EHRs also enable healthcare providers to track patient history, medications, and test results more effectively, leading to more efficient and personalized care. Commentary regarding location specific benefits to the EHR system used may be helpful to further highlight positive aspects of the system.

#Commentary may be useful regarding historical challenges such as cost, technical infrastructure, and data security to support the successful EHR implementation in non-hospital settings/ primary care settings. There are models of EHR care from Australia in rural, remote and First Nations and migrant patients communities including displaced persons that may be useful if expanding on this study with further research. Highlighting strong commentary with collaborative efforts between governments, healthcare providers, and technology companies are essential to overcome these challenges and maximize the benefits of ICT in transforming healthcare delivery.

#For implementing EHRs in non-hospital settings in developing economies is crucial for enhancing healthcare quality and accessibility. By leveraging ICT, healthcare providers can improve patient care, optimize resource allocation, and ultimately, contribute to better health outcomes for all.

#It may be worthwhile providing commentory on how the study has defined the EHR as in terms of globally applicable definitions of EHRs (if they found this was applicable in their research review and if there were any biases flagged for computer processed repository of a patient's health data that enables healthcare specialists to access health records in real-time.

#Specific information that might be helpful to further define for clinical settings to assist in understanding the modular workflows and study reproducibility may be worthwhile to more fully elaborate on patient's health data including demographics, medical conditions, prescribed medications, progress notes thematics, vital signs tracking their physiological parameters, investigations results integrations and network connections with local systems (if applicable) and laboratory data providing diagnostic insights. To replicate the study methodology this has been mentioned but would an exhaustive list of the questionnaire elements be useful?

#Language bias - EHRs offer numerous benefits for both patients and healthcare providers. For patients, EHRs enhance coordination of care by enabling different healthcare providers to access the same information, reducing duplication of tests and procedures, and improving communication between providers.

#In relation to language diversity or patient’s who may have a primary language different to the medical or health care provider examination or commentary on how the system addresses this bias may be useful. One bias with Electronic Health Records (EHR) is that they may not always accurately capture the full picture of a patient's medical history or needs, especially for displaced patients and those from non-English speaking backgrounds. In resource-poor areas, these populations may face additional barriers to accessing and understanding their health information through EHRs.

#Patients also have increased access to their own health information with some EHRs, empowering them to take an active role in managing their health and transferability between health clinics/providers such as some models that exist in nations as Australia. This may be yet to be further developed but considering this as a limitation or comment to further areas of research regarding data ownership/sovereignity.

#Bias / limitations section - Cultural sensitivity and safety framework – uncertain if this exists or has been built into the system as it has been in Australia for First Nations patients. If appropriate identifying if language interpretation services were accessed/is built into the workflows/drop down lists for example as a mechanism to support culturally-sensitive care.

#Identifying in the commentary whether patient confidentiality legislation / workflows are included in the EHR may be useful regarding data governance and data sovereignty for health care which is an expanding global concern for displaced and marginalised communities and identifying “who owns this data”. This may not come into the scope of this study but may be worthwhile considering for future research / modifications if this is an issue for the community involved.

#This study has provided commentary regarding the positive benefits for healthcare providers and patients’ benefit from EHRs by having immediate access to a patient's complete health record, allowing for more efficient and informed decision-making and more streamlined information was very positive and examining some of the intricate issues experiences in this study crafts an understanding of the system for clinicians who have not worked in these conditions. This study also has identified that EHRs also support communication among different healthcare providers, enabling better coordination of care which highlights positive aspects of the system. Additionally, EHRs can help improve patient safety and this commentary in this study has identified the reduction of medical errors was another positive aspect of the study.

Overall, EHRs play a crucial role in modern healthcare by improving the quality, efficiency, and safety of patient care which this study has explored including this study further supporting these types of studies. By enabling comprehensive and real-time access to patient health data, this study has identified the EHRs used to facilitate better coordination, and decision-making among healthcare providers, ultimately leading to improved patient outcomes.

**3. Other points (optional) If applicable, add confidential comments for the editors.**

For readers who may not have an in depth technology and ICT background further exploration or commentary regarding the interventions between NVC and EMA, the EHR and technical difficulties such as the need for syncing multiple times during a patient visit and defining what syncing means and how it impacts the EHR for example if syncing then updates the patients information and saving data, may be useful (from a health literacy perspective).

Biases in relation to cultural biases and how the modular workflows positively address this may be helpful such as how language bias, health literacy and how cultural demographics can affect the patient and clinician experience may be a great future research project to build on this study which could lead to improved patient health outcomes, continuity of care and positive patient and clinician experiences.

If a revised version is done I am happy to support/review this. Important research such as this is commended.
