# Supplementary material for: An offline-first electronic health record for vulnerable populations: A mixed-methods feasibility study
Source: PLOS Digit Health. 2026 Feb 13;5(2):e0001204. doi: 10.1371/journal.pdig.0001204 (PMC12904448; doi:10.1371/journal.pdig.0001204)
Supplement: Attachment — Submitted filename: PLOS GH Response to Reviewers.docx [file pdig.0001204.s007.docx]

**Reviewer #1:**

**Major Issues: None**

**Minor issues**

#Research has primarily focused on barriers at the individual level, and considering if examining the broader organizational/systemic factors and responsibilities for example of the government or how the current health system where the study was conducted uses EHR (or perhaps lack of the EHR) that contribute to the challenges in the health information system identified. There may have been a lack of infrastructure regarding organizational/systemic barriers to allow for prior successful implementation of healthcare technologies in these locations but creating a picture of prior systems or challenges historically may give readers who have not worked in these settings a better understanding. Consider expanding perhaps systemic issues from other nations (deidentifying countries and systemic global commentary) could be helpful in the background section. This may create an opportunity for further local and system health care work/projects to support the patient’s and clinicians and have the study be a vehicle for highlighting any system issues specific to the study location.

For example, regarding biases considering the investment capability in both individual and organizational interventions, may be helpful to communicate the usefulness of the EHR in terms of benefits re: successful implementation of healthcare technologies to highlight global health access issues.

**Response: We appreciate the reviewer's push to have us further discuss the complex nuances that lead to barriers in implementing EHRs. We agree that there are important larger system issues, but the focus of this paper was on clinic-level implementation. We acknowledge the presence of these issues in the introduction (Page 5, Lines 94-96 and 104-107) as well as in the Discussion. We added the specific mention of financial barriers and data security to further emphasize the importance of these factors in line 106.**

#One key advantage of EHRs in non-hospital settings is the ability to centralize patient information, making it easier for healthcare providers to access and share data. This can lead to better coordination of care, reduced medical errors, and improved patient outcomes. EHRs also enable healthcare providers to track patient history, medications, and test results more effectively, leading to more efficient and personalized care. Commentary regarding location-specific benefits to the EHR system used may be helpful to further highlight positive aspects of the system.

**Response: We agree that centralizing patient information is key. This aspect is mentioned in the introduction (Page 5, Lines 101-104, references 6,7), (Page 6, Lines 110-123, references 6,7,10-15), and specific landmark studies are discussed.**

#Commentary may be useful regarding historical challenges such as cost, technical infrastructure, and data security to support the successful EHR implementation in non-hospital settings/ primary care settings. There are models of EHR care from Australia in rural, remote and First Nations and migrant patients communities, including displaced persons that may be useful if expanding on this study with further research. Highlighting strong commentary with collaborative efforts between governments, healthcare providers, and technology companies are essential to overcome these challenges and maximize the benefits of ICT in transforming healthcare delivery.

**Response: We acknowledge the presence of these issues in the introduction (Page 5, Lines 94-96 and 104-107) as well as in the Discussion. We added the specific mention of financial barriers and data security to further emphasize the importance of these factors in line 106.**

**We added additional information about the collaboration of governments, healthcare providers, and software developers in Line 107.**

#For implementing EHRs in non-hospital settings in developing economies is crucial for enhancing healthcare quality and accessibility. By leveraging ICT, healthcare providers can improve patient care, optimize resource allocation, and ultimately, contribute to better health outcomes for all.

**Response: We agree it is crucial and made this an important discussion point throughout the introduction and discussion.**

#It may be worthwhile providing commentory on how the study has defined the EHR as in terms of globally applicable definitions of EHRs (if they found this was applicable in their research review and if there were any biases flagged for computer processed repository of a patient&#39;s health data that enables healthcare specialists to access health records in real-time.

**Response: We have added a specific definition of the HH EHR system to help further clarify (Appendix A). We acknowledge that there are multiple terms used to define systems that catalog health information, including: electronic medical record (EMR), Digital Health Record, Digital Patient Record, and Electronic Patient Record (EPR). From the literature we reviewed and discussed, EHR tends to be a more widely accepted and used term.**

#Specific information that might be helpful to further define for clinical settings to assist in understanding the modular workflows and study reproducibility may be worthwhile to more fully elaborate on patient’s health data including demographics, medical conditions, prescribed medications, progress notes thematics, vital signs tracking their physiological parameters, investigations results integrations and network connections with local systems (if applicable) and laboratory data providing diagnostic insights. To replicate the study methodology this has been mentioned but would an exhaustive list of the questionnaire elements be useful?

**Response: We appreciate the discussion of more specific details on modular workflows and how the HH EHR system works, including how it was built and defined. This has been the focus of prior research and is cited in our manuscript (references 6 and 7). In particular, we provide a more technical development of the HH EHR system. To clarify, we have added a line that specifically mentions this. (Page 7, line 137-138)**

#Language bias - EHRs offer numerous benefits for both patients and healthcare providers. For patients, EHRs enhance coordination of care by enabling different healthcare providers to access the same information, reducing duplication of tests and procedures, and improving communication between providers.

**Response: We agree that there are multiple benefits to improved documentation, which we mention throughout the introduction, results, and discussion.**

#In relation to language diversity or patient’s who may have a primary language different to the medical or health care provider examination or commentary on how the system addresses this bias may be useful. One bias with Electronic Health Records (EHR) is that they may not always accurately capture the full picture of a patient’s medical history or needs, especially for displaced patients and those from non-English speaking backgrounds. In resource-poor areas, these populations may face additional barriers to accessing and understanding their health information through EHRs.

**Response: We agree that a person's primary language is important for understanding and communicating health information. The HH EHR comes with three languages (English, Arabic, and Spanish). We have added this to the introduction (line 107). However, the HH EHR does not currently have a patient-facing interface. Due to this element, we do not think it is worth adding a discussion of this element, even though it is important.**

#Patients also have increased access to their own health information with some EHRs, empowering them to take an active role in managing their health and transferability between health clinics/providers such as some models that exist in nations as Australia. This may be yet to be further developed but considering this as a limitation or comment to further areas of research regarding data ownership/sovereignity.

**Response: We agree that patients having access to their own health information is critical. As we do not have a patient interface at this time and did not make it a focus of this research project, we will not further discuss it at this time. However, this concept has been investigated in other EHR projects (Sijilli) that we do reference and discuss.**

#Bias / limitations section - Cultural sensitivity and safety framework – uncertain if this exists or has been built into the system as it has been in Australia for First Nations patients. If appropriate identifying if language interpretation services wereaccessed/is built into the workflows/drop down lists for example as a mechanism to support culturally-sensitive care.

**Response: In all of the clinics we studied, healthcare providers spoke the primary language patients used. Additionally, this language was used in the EHR system. These details are referenced in prior research (references 6 and 7). We have added a line providing more background information, including this language element on page 7, lines 145-147.**

#Identifying in the commentary whether patient confidentiality legislation / workflows are included in the EHR may be useful regarding data governance and data sovereignty for health care which is an expanding global concern for displaced and marginalised communities and identifying “who owns this data”. This may not come into the scope of this study but may be worthwhile considering for future research / modifications if this is an issue for the community involved.

**Response: We agree that data ownership and adhering to FHIR (Fast Healthcare Interoperability Resources) standards is critical for expanding safe EHR deployment. As we do not own or did we access any patient data for this study, we have opted to not discuss this element.**

#This study has provided commentary regarding the positive benefits for healthcare providers and patients’ benefit from EHRs by having immediate access to a patient’s complete health record, allowing for more efficient and informed decision-making and more streamlined information was very positive and examining some of the intricate issues experiences in this study crafts an understanding of the system for clinicians who have not worked in these conditions. This study also has identified that EHRs also support communication among different healthcare providers, enabling better coordination of care which highlights positive aspects of the system. Additionally, EHRs can help improve patient safety and this commentary in this study has identified the reduction of medical errors was another positive aspect of the study.

Overall, EHRs play a crucial role in modern healthcare by improving the quality, efficiency, and safety of patient care which this study has explored including this study further supporting these types of studies. By enabling comprehensive and real- time access to patient health data, this study has identified the EHRs used to facilitate better coordination, and decision-making among healthcare providers, ultimately leading to improved patient outcomes.

**Response: Thank you for highlighting these important points from our study.**

3. Other points (optional) If applicable, add confidential comments for the editors.

For readers who may not have an in depth technology and ICT background further exploration or commentary regarding the interventions between NVC and EMA, the EHR and technical difficulties such as the need for syncing multiple times during a patient visit and defining what syncing means and how it impacts the EHR for example if syncing then updates the patients information and saving data, may be useful (from a health literacy perspective).

**Response: We believe that the *Practicality* section of the Results goes into a detailed and nuanced discussion of the synchronization between NVC and EMA. As previously mentioned, we reference prior research papers (references 6 and 7) that provide more background on this topic in general.**

Biases in relation to cultural biases and how the modular workflows positively address this may be helpful such as how language bias, health literacy and how cultural demographics can affect the patient and clinician experience may be a great future research project to build on this study which could lead to improved patient health outcomes, continuity of care and positive patient and clinician experiences. If a revised version is done I am happy to support/review this. Important research such as this is commended.

**Response: We agree that cultural bias and patient interaction with EHRs are critical elements that we unfortunately did not explore in this study. We hope to further explore these elements in a true effectiveness trial with an updated version of the HH EHR.**

**Reviewer #2:**

**Major Issues**
1. Lack of thematic justification
The manuscript investigates the feasibility of the Hikma Health Electronic Health Record (HH EHR) across four categories: acceptability, practicality, ease of integration, and effectiveness. However, no clear justification is provided for the selection of these specific themes. The authors should explain why these dimensions were chosen over others, and whether they are based on an established framework, previous literature, or theoretical rationale. Without this, the thematic focus appears arbitrary and limits the interpretability or relevance of the findings.

**Response: Thank you for this specific question on the overall framework we used. We selected four principles out that have been defined in feasibility study frameworks, as they were the most relevant to our innovation and the context in which it was deployed. We based these definitions on the standardly accepted definitions for our specific context. We added a paragraph describing this (Page 8, lines 152-156) with appropriate citations (16 and 17), as well as an appendix with our specific study definitions.(Appendix A)**

2. Contributing factors relevant to clinical and technological characteristics
Another significant omission is the lack of discussion on how previous clinical characteristics, as presented in Table 1, may have influenced participants’ perceptions of the HH EHR. These variables could meaningfully impact user experience and adoption. Additionally, the study does not consider participants’ baseline technological familiarity or digital literacy. Excluding these considerations undermines the validity of the conclusions, particularly those related to ease of use and acceptability

**Response:**

**We agree that the resources that clinics had likely impacted their perspectives of the EHR system. We added a line acknowledging this (Page 12, Lines 230-232).**

**We agree with the reviewer that more detailed information on participants' underlying technology literacy and technology competence are important factors that relate to ease of use and accessibility. We unfortunately did not gather this data, but given the breadth of data we did cover do not think it fully undermines our results. Additionally, prior research on EHR implementation in lower-resource settings has not always gathered this information; see citations 10, 11, 18, and 19 in our manuscript.**

3. Limited discussion on offline EHR system benefits
The discussion does not sufficiently consider the potential advantages of using an offline HH EHR system, even if it lacks the full capabilities of the online solutions. While the limitations of offline functionality are noted, it would strengthen the manuscript to also recognise that offline systems may still provide meaningful benefits, such as improved documentation and continuity of care. Including this perspective would offer a more balanced justification for the implementation of such solutions, especially in low-resource or connectivity-constrained settings.

**Response:**

**We overall believe that we provided a balanced review of our findings, including the benefits of an offline-first system. However, if the impression was that we overemphasized the negative findings, we added additional description of our positive findings in the Discussion section (Page 22, Lines 412-415 ) as well as the Conclusion section (Page 24, Lines 448-453)**

**Minor Issues: None**
